# Engineering the Expression and Characterization of Two Novel Laccase Isoenzymes from *Coprinus comatus* in *Pichia pastori*s by Fusing an Additional Ten Amino Acids Tag at N-Terminus

**DOI:** 10.1371/journal.pone.0093912

**Published:** 2014-04-07

**Authors:** Chunjuan Gu, Fei Zheng, Liangkun Long, Jing Wang, Shaojun Ding

**Affiliations:** Department of Biological Engineering, College of Chemical Engineering, Nanjing Forestry University, Nanjing, Jiangsu, China; Universidade Nova de Lisboa, Portugal

## Abstract

The detail understanding of physiological/biochemical characteristics of individual laccase isoenzymes in fungi is necessary for fundamental and application purposes, but our knowledge is still limited for most of fungi due to difficult to express laccases heterologously. In this study, two novel laccase genes, named *lac3* and *lac4*, encoding proteins of 547 and 532-amino acids preceded by 28 and 16-residue signal peptides, respectively, were cloned from the edible basidiomycete *Coprinus comatus*. They showed 70% identity but much lower homology with other fungal laccases at protein level (less than 58%). Two novel laccase isoenzymes were successfully expressed in *Pichia pastori*s by fusing an additional 10 amino acids (Thr-Pro-Phe-Pro-Pro-Phe-Asn-Thr-Asn-Ser) tag at N-terminus, and the volumetric activities could be dramatically enhanced from undetectable level to 689 and 1465 IU/l for Lac3 and Lac4, respectively. Both laccases possessed the lowest *K*_m_ and highest *k*_cat_/*K*_m_ value towards syringaldazine, followed by ABTS, guaiacol and 2,6-dimethylphenol similar as the low redox potential laccases from other microorganisms. Lac3 and Lac4 showed resistant to SDS, and retained 31.86% and 43.08% activity in the presence of 100 mM SDS, respectively. Lac3 exhibited higher decolorization efficiency than Lac4 for eleven out of thirteen different dyes, which may attribute to the relatively higher catalytic efficiency of Lac3 than Lac4 (in terms of *k*_cat_/*K*_m_) towards syringaldazine and ABTS. The mild synergistic decolorization by two laccases was observed for triphenylmethane dyes but not for anthraquinone and azo dyes.

## Introduction

Laccases [benzenediol: oxygen oxidoreductases (EC1.10.3.2)] are copper-containing enzymes capable of oxidizing a broad spectrum of phenolic compounds and non-phenolic substrates using molecular oxygen as the electron acceptor. In fungi, laccases probably play critical roles in several physiological functions, such as morphogenesis, fungal plant-pathogen/host interaction, degradation of lignocellulosic material, and pigment production [1]. Due to their great versatility and broad substrate specificity, laccases can be used for several industrial applications, such as pulp bleaching in paper industry, textile dye decolorization and detoxification of environmental pollutants [2]. Laccases might also be useful in synthetic chemistry [3]. For instance, laccases have been used to synthesize dyes [4] and products of pharmaceutical importance, such as the anticancer drug mitomycins [5]. The practical applications of laccases in bio-technology would require the suitable laccase for each purpose and economical production of great quantities of pure protein.

In many fungal species, laccases occur as groups of isoenzymes encoded by gene families [6]. For example, in the *Coprinopsis cinerea* genome, 17 nonallelic laccase genes were identified and at least 9 of these members are translated into functional laccase products [7]. Laccase isoenzymes had a highly similar primary structure but differences in expression level and physico-chemical characteristics, thus making difficult to purify individual isoenzymes from fungal cultures for analysis and application. Another approach is the heterologous expression of laccase isoenzymes in a suitable host. Laccase genes have been heterologously expressed in the yeasts *Saccharomyces cerevisiae* and *Pichia pastoris* [8], [9], or filamentous fungi, such as *Trichoderma reesei* [10], and *Aspergillus* spp. [11], [12]. However, notoriously laccases, like other ligninolytic enzymes are relatively difficult to express heterologously in an active form in host contrary to other enzymes. So far, despite the fact that an ever increasing number of laccase gene families has been identified from various fungi by traditional cloning and genome sequencing, only limited number of laccase isoenzymes restricted to few fungi such as *Pleurotus ostreatus*, *Trametes versicolor* and *Lentinula edodes* have been biochemically characterized [13]–[15]. Accordingly, further understanding of precise physiological/biochemical roles of individual laccase isoenzymes in different fungi is necessary for fundamental and application purposes.

*Coprinus comatus* is widely cultivated in many countries as a delicious and highly nutritious edible. The edible mushroom *C. comatus* produced multiple extracellular laccase isozymes, among them, one laccase isoenzyme Lac1 was characterized and exhibited a promising potential in the degradation of some recalcitrant synthetic dyes [16]. The complexity of laccases along with their role in biotechnological applications led us to further investigate other isoenzymes in laccase family of *C. comatus*. In the current study, we successfully expressed two novel laccase isoenzymes in *P. pastori*s by fusing an additional 10 amino acids tag at N-terminus. The biochemical properties and decolorization potentials of two laccase isoenzymes were also compared. The aim of this work was to expand our knowledge of individual laccase isoenzymes in fungi, and in order to identify the new laccase isoenzymes as the candidates for industrial applications.

## Materials and Methods

### Fungal strain, media, and culture conditions

*C. comatus* was provided by the Institute of Edible Fungi, Shanghai Academy of Agricultural Sciences, Shanghai, China, and maintained on potato dextrose agar at 4°C with periodic transfer. *Escherichia coli* DH5a was used as the host for recombinant plasmids. pGEM-T vector (Promega) was used to subclone DNA fragments for sequencing. The vector pPICZαB (Invitrogen) was used for gene expression in *P. pastoris*. All media and protocols for Pichia were according to the Pichia expression manual (Invitrogen). For extraction of RNA, the fungus was grown in stationary 250 ml Erlenmeyer containing 50 ml basal medium with 1 mM caffeic acid at 25°C for 22 days as described previously [17]. Basal medium contained (per liter): 1.0 g KH_2_PO_4_, 0.4 g K_2_HPO_4_, 0.5 g MgSO_4_⋅H_2_O, 0.013 g CaCl_2_⋅2H_2_O, 0.1 g yeast extract, 0.5 g NH_4_NO_3_, 3.0 g asparagine and 2 ml Tween 80. After autoclaving and cooling to room temperature, 2.5 mg/l thiamine and 1 ml/l of a trace-elements solution consisting of (per liter): 4.8 g FeC_6_H_5_O_7_⋅5H_2_O, 2.64 g ZnSO_4_⋅4H_2_O, 2.0 g MnCl_2_⋅4H_2_O, 0.4 g CoCl_2_⋅6H_2_O and 0.4 g CuSO_4_⋅5H_2_O was added. Four agar discs (1 cm diameter), cut from the growing edge of a 7-day PDA culture, were used to inoculate each flask.

### Cloning of laccase isoenzymes *lac3* and *lac4*

The genomic DNA was extracted from *C. comatus* with the method described by Hoshida et al. [18]. Total RNA was isolated from 22-day-old mycelia using a TRizol reagent (Invitrogen) according to the manufacturer instructions and used for cDNA synthesis. Degenerate primers LacCu1 and LacCu2 (Table 1) were designed according to the conserved sequences of the copper-binding regions I (HWHGFFQ) and II (GTFWYHS) in most fungal laccases, respectively. PCR was carried out using Taq DNA polymerase (Takara), and the genomic DNA as the template. The PCR reaction programme was initiated at 94°C for 5 min, followed by 30 cycles of 94°C for 30 s, 55°C for 30 s and 72°C for 2 min, and a final extension at 72°C for 10 min. The 200 bp PCR products were cloned into the pGEM-T vector and 12 randomly selected clones were sequenced. The nucleotide sequences of the 12 clones were classified into 3 groups. Among them, one was identical with the previously reported *lac2*, two were new laccase isoenzymes, designed as *lac3* and *lac4*, respectively. The 5′-end cDNA fragments were amplified by RACE-PCR using the SMART RACE cDNA Amplification Kit (Clontech) and primers Lac3 GSP1 and Lac4 GSP1 for *lac3* and *lac4*, respectively. The fragments were subcloned into pGEM-T vector and sequenced. The full-length cDNAs of *lac3* and *lac4* were then generated by 3′-RACE using the gene-specific primers Lac3GSP2 and Lac4GSP2 designed from the sequence of the extreme 5′-cDNA ends of *lac3* and *lac4* respectively, and sequenced as above. Phylogenetic analyses were performed using the MEGA version 5.0 software [19]. Sequences were aligned globally using the Clustal W program in MEGA. Trees were constructed by the neighbor-joining method with a Poisson correction model.

**Table 1:** Sequences of primers used in this study.

Primers	Sequences	Purpose in this study
LacCu1	5′-CAY TGG CAY GGN TTY TTY CA-3′	Gene fragment
LacCu2	5′-G RCT GTG GTA CCA GAA NGT NCC-3′	Gene fragment
Lac3 GSP1	5′-GGACATTGATTGACACCAGTCGCGCCA-3′	5′-RACE of lac3
Lac4 GSP1	5′-CTCCGGAACCACCATCAGCCCAATTAG-3′	5′-RACEof lac4
Lac3 GSP2	5′-CTCACGACCTCCCGCTTGGGAATAACCAAC-3′	3′-RACEof lac3
Lac4 GSP2	5′-GGCGCCAATGCTTTTGCGCTTTCTGTCGG-3′	3′-RACEof lac4
Lac3F	5′-CGGAATTCTCAGTCCTCATACCGCACTCGAC-3′	pPICZαB-Lac3,
Lac3R	5′-GCTGGCGGCCGCAACTGGCATTGGTAAAGAAGAGACA-3′	pPICZαB-Lac3,
Lac4F	5′-CGGAATTCCTTTCTGTCGGTCCACGAGCAAC-3′	pPICZαB-Lac4
Lac4R	5′-GCTGGCGGCCGCATGTTTCACCATAGGCACAAT-3′	pPICZαB-Lac4
pPICZαB-10AAF	5′-GATCTAGAAGAGTTGGTGTTGAAAGGGGGGAATGGCGTCGGAATTCCTGCAGCTTCAGCCT-3′	pPICZαB-10AA
pPICZαB-10AAR	5′-TTCCGCGGGAACAAAAACTCATCT-3′	pPICZαB-10AA
Lac310AAF	5′-GCTCTAGATCAGTCCTCATACCGCACTCGAC-3′	pPICZαB-10AALac3
Lac310AAR	5′-GTCCCCGCGGAACTGGCATTGGTAAAGAAGAGACA-3′	pPICZαB-10AALac3
Lac410AAF	5′-GCTCTAGACTTTCTGTCGGTCCACGAGCAAC-3′	pPICZαB-10AALac4
Lac410AAR	5′-GTCCCCGCGGATGTTTCACCATAGGCACAAT-3′	pPICZαB-10AALac4
Lac3mycR	5′-GTCCCCGCGGTCAATGATGATGATGATGATGAACTGGCATTGGTAAAGAAGAGACA-3′	pPICZαB-10AALac3-ΔMyc
Lac4mycR	5′-GTCCCCGCGGTCAATGATGATGATGATGATGATGTTTCACCATAGGCACAAT-3′	pPICZαB-10AALac4-ΔMyc

In the degenerate primers the following abbreviations were used Y = C, T; N = A, G, C, T; M = C, A; R = A, G; V = G, A, C.

### Construction of expression plasmids

**Figure 1:**
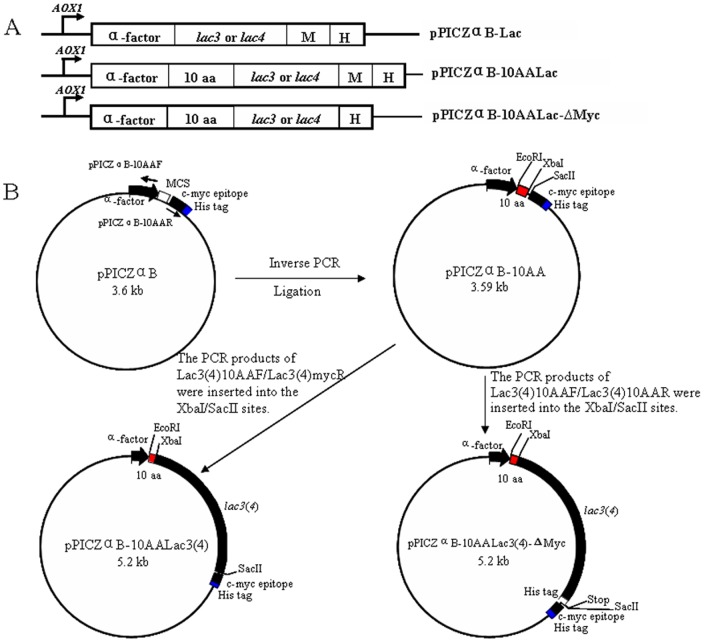
Schematic representation of constructs for engineering the expression of *lac3* and *lac4* (A) and strategy for the construction of expression plasmids pPICZαB-10AALac3, pPICZαB-10AALac4, pPICZαB-10AALac3-ΔMyc and pPICZαB-10AALac4-ΔMyc (B). M: C-terminal myc epitope; H: polyhistidine tag

Six different expression plasmids were constructed in order to functionally express *lac3* and *lac4* in *P. pastoris*, (Fig. 1). For the constructs with mature laccase genes, the *lac3* and *lac4* genes were PCR-amplified using primer pairs Lac3F/Lac3R, and Lac4F/Lac4R respectively. After double digestion with EcoR I and Not I, the fragments were ligated with the same digested vector pPICZαB in frame with the C-terminal myc epitope and the polyhistidine tag to yield the constructs, pPICZαB-Lac3 and pPICZαB-Lac4. For expression of laccase genes containing an additional N-terminal 10 amino acids tag, the expression vector pPICZαB-10AA was generated by PCR using primer pair pPICZαB-10AAF/pPICZαB-10AAR and template pPICZαB. In which the phenylalanine and proline rich motif Thr-Pro-Phe-Pro-Pro-Phe-Asn-Thr-Asn-Ser (TPFPPFNTNS), derived from the first N-terminal 10 amino acids residues of the mature xylanase (XynII) in *V. volvacea* [20], was fused with pPICZαB at EcoRI site. Then the *lac3* and *lac4* genes were PCR-amplified using primer pairs Lac310AAF/Lac310AAR and Lac410AAF/Lac410AAR respectively. Both of the amplified pPICZαB-10AA and gene products were flanked by Xba I and Sac II restriction sites at the 5′- and 3′-ends, respectively. After double digestion with XbaI and Sac II, the fragments were ligated with the same digested pPICZαB-10AA in frame with the C-terminal myc epitope and the polyhistidine tag to yield the constructs, pPICZαB-10AALac3 or pPICZαB-10AALac4. For the constructs without Myc tag, the *lac3* and *lac4* genes with 6×His tag followed by a termination codon at C-terminus were PCR-amplified using primer pairs Lac310AAF/Lac3mycR and Lac410AAF/Lac4mycR respectively. The fragments were inserted into the pPICZαB-10AA after digested with Xba I and Sac II to yield plasmids pPICZαB-10AALac3-ΔMyc and pPICZαB-10AALac4-ΔMyc. All the cDNA constructs were verified by DNA sequence analyses. PCR conditions were: one cycle of 94°C for 5 min, 55°C for 30 s and 72°C for 3 min; 30 cycles of 94°C for 30 s, 55°C for 30 s and 72°C for 3 min followed by a final extension at 72°C for 5 min.

### Expression of laccase isoenzymes *lac3* and *lac4*

All the expression plasmids were linearized with SacI and transformed into *P. pastoris* KM71H competent cells by electroporation with a Genepulser II apparatus (Bio-Rad, Hercules, CA). Transformants containing the *lac3* and *lac4* cDNA were selected on yeast extract-peptone-dextrose (YPD) agar plates with 1 M sorbitol and 100 μg/ml Zeocin (Invitrogen). The candidate transformants were transferred to minimal methanol (MM) plates containing 0.2 mM ABTS and 0.1 mM CuSO_4_. Laccase-producing transformants were identified by the presence of dark green color appearance around the colonies after four days growth. The high expression transformants which showing a deeper color in the plate were selected for liquid fermentance experiment. The transformed yeast cells were grown in 250 ml flasks containing 50 ml BMGY medium at 250 rpm and 30°C. After the OD_600_ value reached 6, cells were harvested by centrifugation, and resuspended in 15 ml of BMMY medium containing 500 μM CuSO_4_ in 250 ml Erlenmeyer flasks and induced for a further 15 days by adding methanol to a final concentration of 0.5% at 24 h intervals. Every 24 h, 0.5 ml of cultures was sampled from the flasks and laccase activity was determined after separating yeast cells by centrifugation.

### Enzyme activity assay

The routine assay for laccase activity towards 2,2′-azino-bis-[3-ethylthiazoline-6-sulfonate] (ABTS) as the substrate was measured in 1 ml reaction volume containing 1 mM ABTS, 100 mM sodium acetate buffer (pH 4.5) and 5 μl aliquots of appropriately diluted enzyme sample. Oxidation of ABTS was monitored by following the increment in A 420 (ε_420_ = 3.6×10^4^ M^−1^cm^−1^) [21]. One unit of laccase activity was defined as the amount of enzyme required to oxidize 1 μmol ABTS min^−1^ at 40°C. The activities towards syringaldazine and 2,6-dimethylphenol were assayed under same condition as above, oxidation of syringaldazine and 2,6-dimethylphenol was followed by an absorbance increase at 525 nm (ε_525_ = 6.5×10^4^ M^−1^.cm^−1^) and 421 nm (ε_421_ = 5.78×10^4^ M^−1^.cm^−1^), respectively. The activity towards guaiacol was measured in 2.4 ml reaction mixture containing 1 mM guaiacol, oxidation of guaiacol was monitored by an absorbance increase at 465 nm (ε_465_ = 1.21×10^4^ M^−1^.cm^−1^) after 30 min reaction. Protein concentration was determined by a BCA Protein Assay Kit (Thermo Scientific Pierce) with bovine serum albumin as standard.

### Purification and characterization of recombinant Lac3 and Lac4

Culture supernatants from 500 ml BMMY cultures were collected by centrifugation (5,000 g for 15 min) and concentrated <20-fold with the Pellicon ultrafiltration system (Millipore) using a 10 kDa molecular mass cut-off membrane. The concentrated enzyme solution was dialyzed overnight against 20 mM Tris–HCl (pH 8.0) and any remaining precipitate was removed by centrifugation at 10,000 g for 30 min. The supernatant was applied to a DEAE Sepharose CL-6B (1.6×50 cm, Dingguo, Beijing, China) pre-equilibrated with the same buffer. After washed with same buffer to remove unbound proteins, the bound laccases were subsequently eluted from the column with a linear salt gradient (0–1.0 M NaCl) in the same buffer. The active fractions were pooled, concentrated to<5 ml by ultrafiltration and further purified by fast protein liquid chromatography (FPLC) gel filtration on a Superdex 75 HR column (Tricorn-10/600 47.5 ml, GE Healthcare) in the same buffer (pH 7.5) using an AKTA Purifier (GE Healthcare). The combined active fractions were concentrated to <3 ml by ultrafiltration as described above, and stored at −20°C for further use. Enzyme homogeneity and the molecular weight of purified Lac3 and Lac4 were estimated using sodium dodecyl sulfate–polyacrylamide gel electrophoresis (SDS-PAGE) (10% w/v) and the protein molecular weight markers (TaKaRa) as a reference under reducing conditions. Optimal pH and temperature values were determined using the ABTS, guaiacol, 2,6-dimethylphenol and syringaldazine (each at 1 mM) as substrates over the ranges pH 1.0–9.0 (100 mM KCl–HCl buffer pH 1.0–2.0, universal buffer: 50 mM H_3_PO_4_, 50 mM CH_3_ COOH, 50 mM H_3_BO_3_, pH 2.0–9.0 adjusted with 0.2 M NaOH at 25°C) and 30–80°C, respectively. The thermal stability was determined by measuring the residual activities with substrate ABTS after pre-incubation of the enzymes at 30°C to 70°C for 0–60 min. The kinetic constants (*V*_max_ and *K*_m_) were respectively determined by measuring the rates of substrates oxidation using substrate concentration ranges of 0.3–1.0 mM, 0.06–1 mM, 0.3–1.0 mM, and 0.08–0.6 mM for ABTS, guaiacol, 2,6-dimethylphenol and syringaldazine at their optimal temperature and pH value. The pH values for substrate specificity and kinetic constants measures were 3.0, 5.0, 5.5, and 5.0 for Lac3, and 3.0, 6.0, 5.5 and 6.0 for Lac4 for ABTS, guaiacol, 2,6-dimethylphenol and syringaldazine, respectively. The *V*_max_ and *K*_m_ values were calculated by Graphpad Prism 5.0 software (http://www.graphpad.com/prism/) using nonlinear regression. The effect of various metal ions (each at 1 mM) and inhibitors (EDTA, SDS, each at 5–100 mM; DTT, 0.001–1 mM) on Lac3 and Lac4 activity was assessed by pre-incubating the purified enzyme (0.2 IU/ml) with different metal ions and inhibitors at 4°C for 30 min. The residual activity was determined using ABTS as a substrate under the standard assay conditions.

### Deglycosylation of recombinant Lac3 and Lac4

Purified recombinant Lac3 and Lac4 were treated with endoglycosidase H (endo- N-acetylglucosaminidase H of *Streptomyces plicatus*; NEB) according to the manufacturer's protocol. Lac3 or Lac4 (2 μg each) in 1× glycoprotein denaturing buffer was boiled for 10 min. After cooling, 1 μl of endoglycosidase H was added and the sample was incubated at 37°C overnight. The product of endoglycosidase H treatment was analyzed by SDS-PAGE.

### Dyes decolorization

Thirteen different dyes were used for this study. The reaction mixture (2 ml) contained 100 mM acetate buffer pH 4.5, individual dye (each 50 mg/l in final concentration), and 0.5 IU laccase with or without a redox mediator 1-hydrox-ybenzotriazol (HBT, final concentration 0.1 mg/ml). The reaction was initiated by the addition of laccase and incubated at 40°C for 12 h. For synergistic decolorization by two laccases, Lac3 and Lac4 (0.25 IU each) were added in reaction mixture. Decolorization was determined by monitoring the decrease in absorbance at the peak of maximum visible absorbance and expressed as percentage of decolorization. Decolorization was defined as: Decolorization (%)  = 100×(*Ao* - *At*)/*Ao*. Where *Ao* is the absorbance of the reaction mixture before incubation with the enzyme and *At* is absorbance after incubation. The heat-denatured laccase solutions were used as controls and the blanks contained all components of the reaction mixture except the dyes.

### Nucleotide sequence accession numbers

The nucleotide sequences of *lac3* and *lac4* were deposited in the European Nucleotide Archive (ENA) under accession numbers HG764548 (*lac3*) and HG764549 (*lac4*) respectively.

## Results

### Cloning and sequence analysis of *lac3* and *lac4*

**Figure 2:**
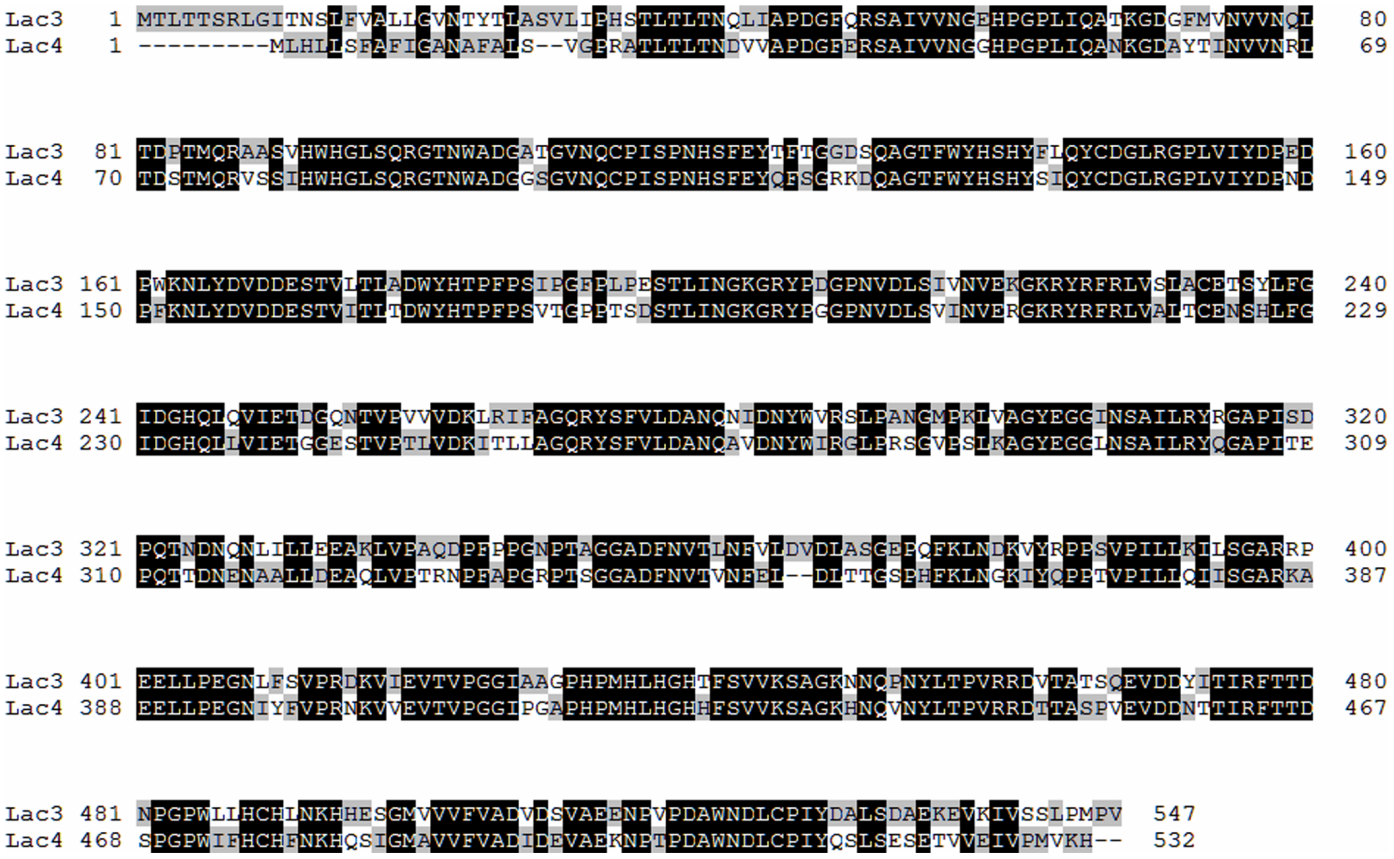
Alignment of deduced amino acid sequences of *lac3* and *lac4*.

Two cDNAs encoding new laccase isoenzymes Lac3 and Lac4 in *C. comatus* were cloned in this study. As predicted from the full-length cDNA clones, *lac3* and *lac4* encode 547- and 532-aa-long polypeptides with a putative signal peptide of 28 or 16 amino acids, respectively. They show 70% amino acid pairwise identity (Fig. 2), and each contains 3 potential N-glycosylation sites. The calculated pI values of predicted mature Lac3 and Lac4 are 5.16 and 5.8, respectively. The deduced amino acid sequences of *lac3* and *lac4* were compared with the sequence of other laccases available in the GenBank database. *Lac3* was most closely related to *Laccaria bicolor* laccase (XP_001881441.1) and *Coprinopsis cinerea* laccase 4 (XP_001829763.2) with a 57% identity, followed by *Lentinula edodes* laccase (AET86511.1), *Coprinopsis cinerea* laccase 8 (AAR01249.1) and laccase 1 with 55%, 53% and 53% identities respectively. Whereas *lac4* exhibited the maximum identity (58%) with *Laccaria bicolor* laccase (XP_001881441.1), followed by *Coprinopsis cinerea* laccase 4(XP_001829763.2), laccase 8 (AAR01249.1) and laccase 1 (AAS38574.1), and *Cyathus bulleri* laccase (ABW75771.2) with 56%, 56%, 55%, 54%, and 54% identities respectively. A phylogenetic analysis of protein sequences revealed that *lac3* and *lac4* belonged to the same phylogenetic groups (Fig.S1).

### Heterologous expression of *lac3* and *lac4* in *Pichia pastoris*

**Figure 3:**
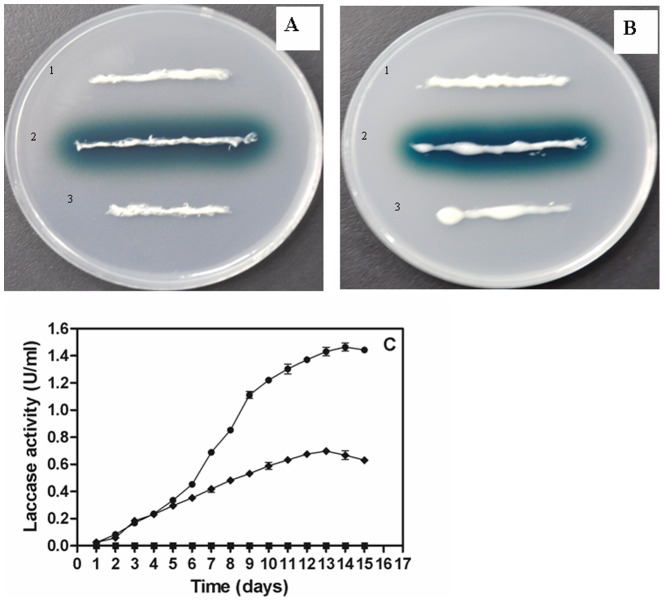
Detection of laccase activity on BMMY agar plates containing CuSO_4_ and ABTS for transformants with *lac3* gene (A) and *lac4* gene (B) and the time course of extracellular laccase production in BMMY liquid medium (C). A: transformants containing 1) pPICZαB-Lac3, 2) pPICZαB-10AALac3, 3) pPICZαB-10AALac3-ΔMyc; B: transformants containing 1) pPICZαB-Lac4, 2) pPICZαB-10AALac4, 3) pPICZαB-10AALac4-ΔMyc; C: transformants containing pPICZαB-10AALac3 (filled diamond), pPICZαB-10AALac4 (filled circle), pPICZαB-Lac3, pPICZαB-Lac4, pPICZαB-10AALac3-ΔMyc and pPICZαB-10AALac4-ΔMyc (filled square).

To express the *lac3* and *lac4* in *P. pastoris*, six different expression plasmids were constructed under the control of the tightly regulated AOX1 promoter. By the plate detection, the dark green zones appeared around the transformants containing constructs pPICZαB-10AALac3 or pPICZαB-10AALac4, carrying the fusion laccase genes with an additional N-terminal 10 aa tag in frame with the C-terminal myc epitope and the polyhistidine tag (Fig. 3A and 3B). The results implied that bioactive Lac3 and Lac4 were expressed and secreted into the extracellular medium. On the contrary, no dark green zones appeared around the transformants containing constructs pPICZαB-Lac3, pPICZαB-Lac4, pPICZαB-10AALac3-ΔMyc or pPICZαB-10AALac4-ΔMyc, demonstrating the essential role of the additional 10AA tag at N-terminus and myc epitope at C-terminus in *lac3* and *lac4* expressions in *P. pastoris*. The laccase-positive transformants with constructs pPICZαB-10AALac3 or pPICZαB-10AALac4, as well as the laccase-negative transformants, were then fermented in BMMY liquid medium at 30°C and induced by addition of 0.5% (v/v) methanol daily. After 14-days growth, the laccase activities reached 689 and 1465 IU/l for Lac3 and Lac4 respectively (Fig. 3C). No extracellular laccase activity was detected in culture supernatants of the laccase-negative transformants containing pPICZαB-Lac3, pPICZαB-Lac4, pPICZαB-10AALac3-ΔMyc or pPICZαB-10AALac4-ΔMyc respectively. *Lac2* gene, which could be not expressed in *P. pastoris* [16], was used to assess whether the N-terminal 10 aa tag is effective for other laccases expression. The green zone appeared around the transformant containing plasmid pPICZαB-10AALac2, carrying the fusion *lac2* gene with the N-terminal 10 aa tag in frame with the C-terminal myc epitope and the polyhistidine tag (Fig. S2A). The laccase activity reached 60.1 U/l after 14-days growth at 30°C (Fig. S2B).

### Purification and characterization of recombinant Lac3 and Lac4

**Figure 4:**
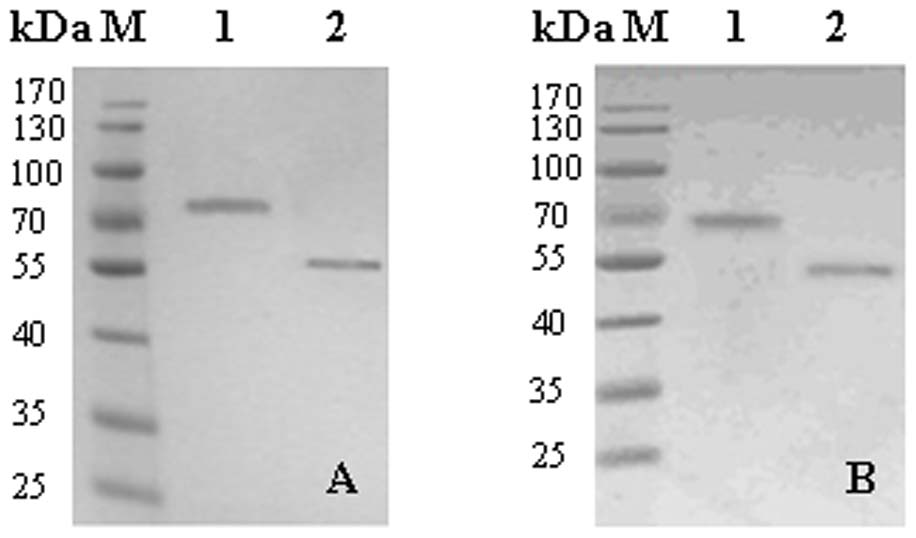
SDS-PAGE of recombinant Lac3 (A) and Lac4 (B). A: M, Protein markers, lane 1, purified Lac3, lane 2, endoglycosidase-H-treated Lac3; B: M, Protein markers, lane 1, purified Lac4, lane 2, endoglycosidase-H-treated Lac4.

**Figure 5:**
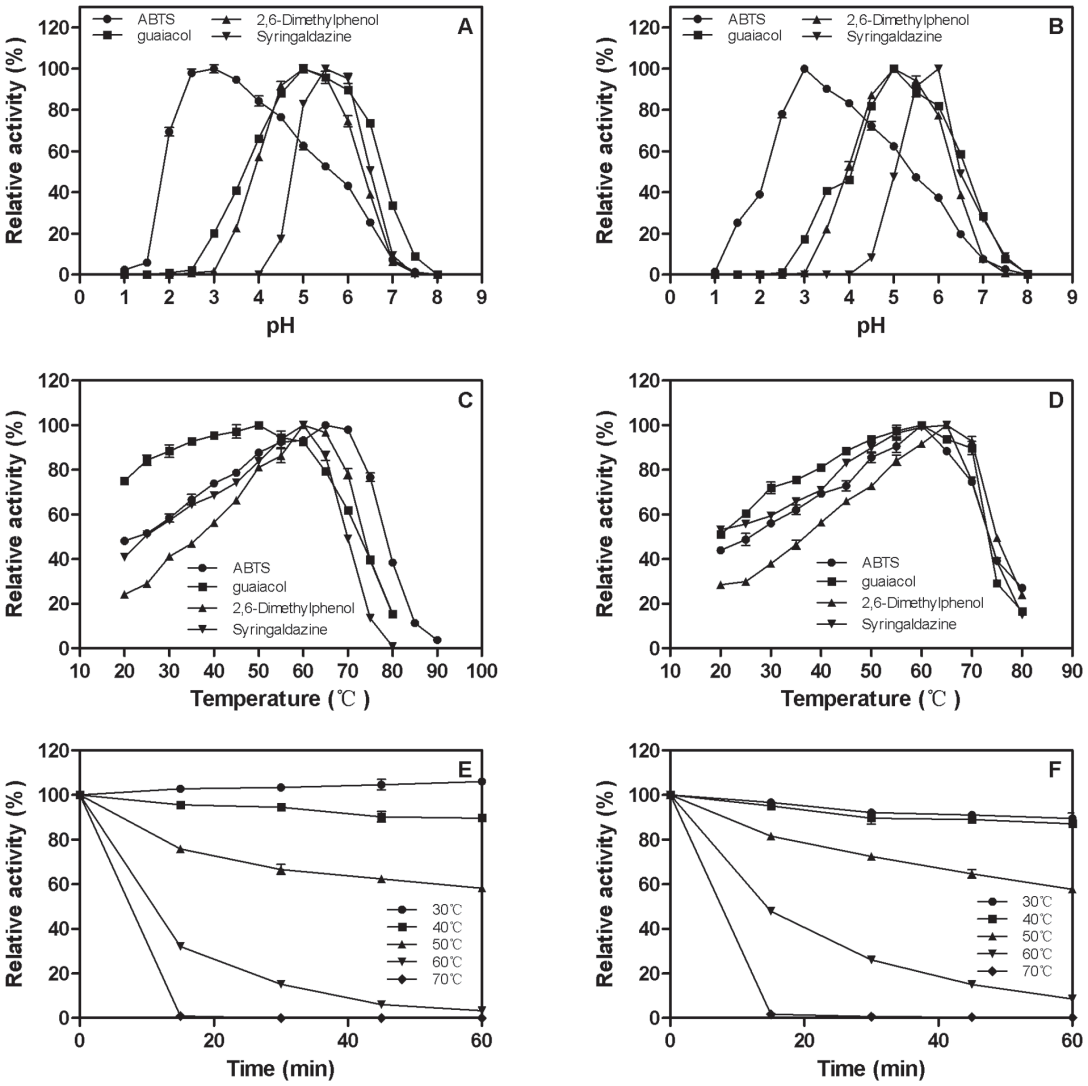
Effects of pH on the activity of Lac3 (A) and Lac4 (B), effects of temperature on the activity of Lac3 (C) and Lac4 (D) and effects of temperature on the stability of Lac3 (E) and Lac4 (F). Filled diamond, ABTS; filled square, guaicol; filled triangle, 2,6- Dimethylphenol; and filled circle, Syringaldazine. The values are the mean of triplicate experiments ± SD.

The recombinant Lac3 and Lac4 were purified by chromatography with two steps procedure. SDS-PAGE analysis revealed that the molecular masses of purified recombinant Lac3 and Lac4 were about 75 kDa and 70 kDa, which was higher than the predicted masses of 54 and 53 kDa, respectively (Fig. 4A and 4B). After treatment with endoglycosidase H (endoH), the molecular masses of Lac3 and Lac4 were reduced to 55 kDa and 52 kDa respectively (Fig. 4A and 4B), indicating the recombinant laccases were glycosylated. The optimal pH values of Lac3 or Lac4 were 3.0 for ABTS, 5.5 for syringaldazine, 5.0 or 6.0 for guaiacol, and 5.0 or 6.0 for 2,6-dimethylphenol, respectively (Fig. 5A and 5B). Meanwhile, the optimal temperature values of Lac3 or Lac4 were 65°C or 60°C for ABTS, 60°C or 65°C for syringaldazine, 50°C or 60°C for guaiacol, and 60°C or 65°C for 2,6-dimethylphenol, respectively (Fig. 5C and 5D). The recombinant laccases were stable at temperatures below 50°C (Fig. 5E and 5F), and were very sensitive to DTT but not to EDTA and SDS (Table 2). Lac3 and Lac4 showed high resistant to SDS, and retained 31.86% and 43.08% activity in the presence of 100 mM SDS, respectively. The metal ions such as Ca^2+^, Mg^2+^, Co^2+^, Zn^2+^, Mn^2+^ and Cu^2+^ had slight or even no effect on the activities of Lac3 and Lac4 (Table 2). Kinetic parameters of the laccases were determined by using ABTS, guaiacol, 2,6-dimethylphenol and syringaldazine as substrates and summarized in table 3. Remarkably, two laccases demonstrated different substrate affinities and turnover rates (*k*_cat_). Lac3 had significantly lower *K*_m_ and higher *k*_cat_/*K*_m_ values than Lac4 towards ABTS, syringaldazine and 2,6-dimethylphenol.

**Table 2:** Effects of metal ions and inhibitors on Lac3 and Lac4 activity.

		Relative activity (%)	
Inhibitor	Concentration (mM)	Lac3	Lac4
None	–	100	100
EDTA	5	93.02	98.14
	10	88.29	83.85
	20	57.97	41.42
	50	29.71	20.00
	100	0	0
SDS	5	74.55	89.73
	10	61.49	74.06
	20	54.19	60.59
	50	47.13	48.58
	100	31.86	43.08
DTT	0.001	96.65	90.79
	0.01	85.91	75.95
	0.1	1.59	0.83
	1	0	0
Ca2+	1	83.43	94.61
NH4+	1	102.83	98.17
Mg2+	1	95.97	101.45
Co2+	1	98.54	93.93
Zn2+	1	97.68	91.43
Ni+	1	106.35	101.64
Na+	1	100.17	92.20
Mn2+	1	92.10	91.43
K+	1	95.88	97.69
Cu2+	1	93.65	100

Values are the mean of triplicate determinations and standard deviation is less 5%.

**Table 3:** Kinetic parameters for recombinant Lac3 and Lac4.

			*K*_m_ (mM)		*k*_cat_ (s^−1^)		*k*_cat_/*K*_m_ (s^−1^mM^−1^)	
Substrates	Wavelength (λ max, nm)	Molar Extinction Coefficient (M^−1^cm^−1^)	Lac3	Lac4	Lac3	Lac4	Lac3	Lac4
Syringaldazine	525	65000	0.087	0.204	31.96	44.71	367.36	219.60
ABTS	420	36000	0.136	0.205	3.485	1.467	25.625	7.167
Guaiacol	465	12100	1.114	0.455	0.418	0.196	0.375	0.503
2,6-Dimethylphenol	421	57800	1.374	7.131	0.307	1.468	0.223	0.206

### Dye decolorization by recombinant Lac3 and Lac4

**Figure 6:**
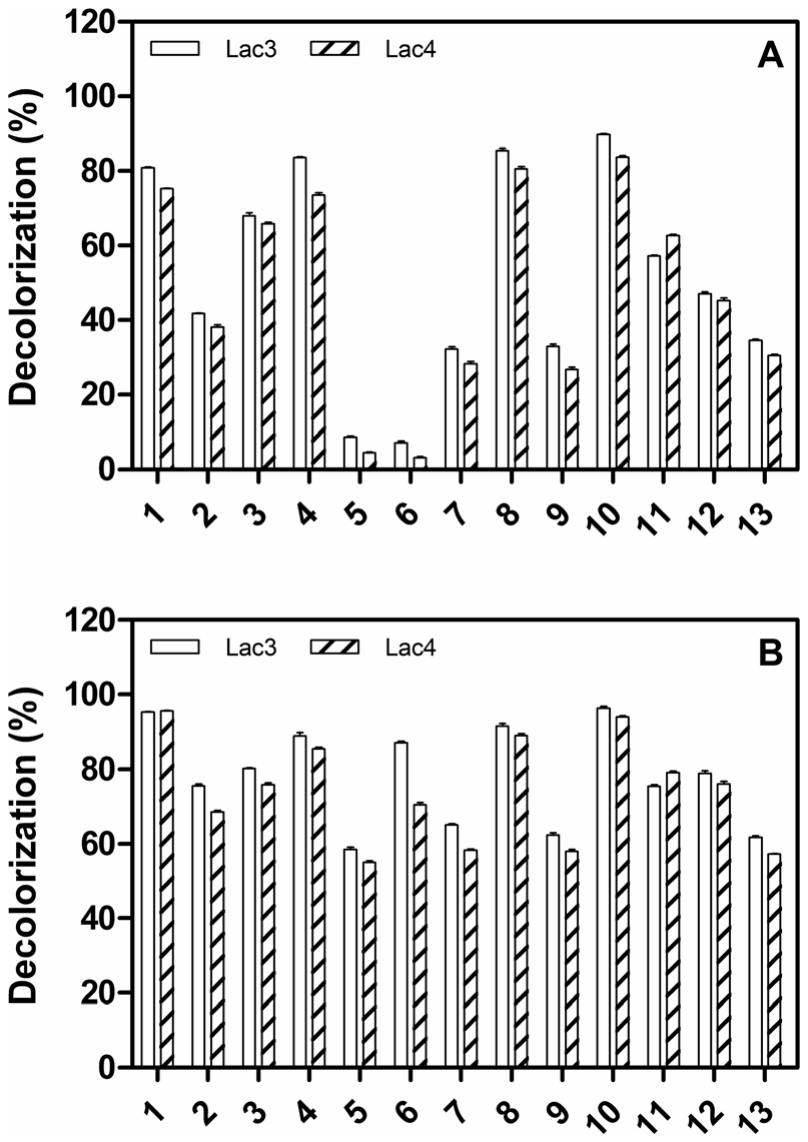
Decolorization of synthetic dyes by Lac3 or Lac4 only (A) and in the presence of HBT (B). 1) Remazol brilliant blue R, 2) Reactive brilliant blue X-BR, 3) Reactive brilliant blue K-GR, 4) Reactive brilliant blue K-3R, 5) Reactive orange 1, 6) Reactive red X-3B, 7) Congo red, 8) Reactive dark blue KR, 9) Coomassie G-250, 10) Malachite green, 11) Bromophenol blue, 12) Methyl violet,13) Victoria blue B. The values are the mean of triplicate experiments ± SD.

Thirteen synthetic dyes including anthraquinone, azo and triphenylmethane dyes were used to evaluate the decolorization ability of the recombinant Lac3 and Lac4 with or without HBT. Among the three types of dyes, the recombinant Lac3 and Lac4 showed higher decolorization efficiency for anthraquinone than triphenylmethane and azo dyes in the absence of HBT (Fig. 6A). In the presence of HBT, the decolorization efficiencies of Lac3 respectively increased to 95.34%, 91.7%, and 96.33% for Remazol brilliant blue R, Reactive dark blue KR and Malachite green after 12 h of incubation, while the corresponding values by Lac4 were 95.66%, 89.12% and 93.96% respectively (Fig. 6B). The other dyes were decolorized to different extend within 12 h as revealed in Fig. 6. The broad decolorization specificity of Lac3 and Lac4 rendered their great potentials in industrial applications, such as degradation of dyes from textile effluents. It was interesting that Lac3 showed higher decolorization ability than Lac4 towards most of dyes with except of Remazol brilliant blue R and Bromophenol blue. The mild synergistic decolorization by two laccases was observed for triphenylmethane dyes, and the decolorization percentages were enhanced by 5% when mixed laccases used in reaction comparing with Lac3 or Lac4 alone. But no synergistic decolorization was detected for anthraquinone and azo dyes (data not shown).

## Discussion

Two cDNAs encoding novel laccase isoenzymes in *C. comatus* were identified in this study. They showed 70% identity but lower homology with previously cloned Lac1 and Lac2 from *C. comatus* and other fungal laccases. At protein level, Lac3 and Lac4 have 50% and 47.2% identities with the Lac1, and 51.9% and 50.6% identities with the Lac2, respectively. Furthermore, unlike some high redox potential fungal laccases bearing a Leu or Phe residue at the amino acid residue 10 aa downstream of the conserved cysteine respectively, Lac3 and Lac4 have non-coordinating Met at this position. According to the current literatures, several structural features may contribute to the redox potential of laccases, including the amino acids which form the T1 pocket and the coordination sphere of the T1 copper [22], [23]. Lac3 and Lac4 are provisionally grouped into low redox potential classes based on axial ligand type (Met as axial ligand), as similar with other low redox potential bilirubin oxidase from ascomycete *Myrothecium verrucaria* [24], laccase from plant *Rhus vernicifera* [25] and archaeal laccase from *Haloferax volcanii* [26]. The simultaneous presence of high redox and low redox potential laccases has been reported in *Trametes* sp. C30 [27], [28].

Various yeast and filamentous fungal species have been used as hosts to heterologously express laccase genes for their characterization and application. For example, different laccase isoenzymes from *Trametes versicolor* were successfully expressed in yeasts *Yarrowia lipolytica* [29], *Pichia methalonica* [30]. *P. pastoris* [13], [31] and *Saccharomyces cerevisiae* [32] or filamentous fungi *Aspergillus niger* [33] and *Trichoderma reesei* [34]. The *P. pastoris* system may still be the most frequently used host owing to convenience and speed, although the expression levels was considerably lower than some yeasts and filamentous fungi. Over 20 fungal laccases have been heterologously expressed in *P. pastoris* for different purposes [14]. However, the production remained low and even no expression for some laccases. The inability of yeasts to process the post-translation of different laccase genes with the same efficiency may explain the observed “selectivity” in expression [14]. There are several strategies used to increase the expression level of heterologous proteins in *Pichia* [35], such as the use of native promoters and multiple gene copies, codon optimization, altering of secretory signal sequences, and optimization of culture conditions. However, these were not successful in some cases for fungal laccase expression [3]. In this study, we successfully expressed two laccase isoenzymes in *P. pastoris* by fusing the genes with the N-terminal 10 aa tag from the xylanase of *V. volvacea* [20]. The successful expression of *lac2* by fusing the additional 10 aa tag further confirmed the effective of this tag in improving laccase expression in *P. pastor*is. The reason for the great increase in the expression levels was unclear. It has been reported in literuture that the deletion or added N-terminal amino acids and C-terminal sequence were beneficial for increasing the protein secretion levels in *P. pastoris* [36], [37]. It can be safely assumed that the additional N-terminal 10 aa peptide together with C-terminal myc epitope may contributes to the correct post-translationally processing of Lac3 and Lac4 in *Pichia*. Our data suggested that the additional phenylalanine and proline rich motif could be useful tag for increasing heterologous protein expression in yeast.

When laccases were expressed in *P. pastoris*, the recombinant laccases were found be hyperglycosylated. However, no reports show that hyperglycosylation affects the activity of the enzyme produced [33]. By the molecular mass determination before and after endo-H deglycosylation, glycosylation patterns of the recombinant Lac3 and Lac4 was 36.4% and 34.6% respectively, similar as other fungal laccases expressed in *P. pastoris* [1]. Two laccases in general showed low optimum pH for ABTS, but relative higher for other substrates similar as other fungal laccases [1]. Lac4 has higher optimal pH values than Lac3 when assayed with guaiacol and 2,6-dimethylphenol. Lac4 also exhibited higher optimal temperature than Lac3 for guaiacol, syringaldazine and 2,6-dimethylphenol. Although they demonstrated obvious differences in substrate affinities and turnover rates (*k*cat), both laccases possessed the lowest *K*_m_ and highest *k*_cat_/*K*_m_ value towards syringaldazine, followed by ABTS, guaiacol and 2,6-dimethylphenol. From these findings, it is obvious that Lac3 and Lac4 were clearly distinguishable from the previously cloned Lac1 from *C. comatus* [16] and other well known fungal laccase [1], but possessed similar biochemical properties as the low redox potential laccases from *Melanocarpus albomyces* [38] and *Trametes* sp. C30 Lac2 and Lac3 [27], [28], or archaeal laccase from *Haloferax volcanii* [26]. Sodium dodecyl sulphate (SDS) is a strong protein denaturant that inactivates most laccases even at a low concentration [39], [40]. Lac3 and Lac4 displayed remarkably higher SDS resistance than many of published fungal laccases, thus showing an application potential in detergent-containing conditions.

Laccase-mediated dye decolorization has been described with crude or purified forms from many fungal species. The decolorization rate varied according to the source of enzymes and the structures of different dyes. The decolorization capability of Lac3 and Lac4 were similar or even higher than many reported fungal laccases. For example, it was reported that the laccases derived from *P. ostreatus* and *Ganoderma lucidum* could decolorize 70% and 40.7% of malachite green (50 mgl^−1^) after 24 h of incubation [41], [42]. In our study, the decolorization efficiencies reached 89.73% and 83.73% for Lac3 and Lac4 after 12 h of incubation without redox mediator respectively. Lac3 exhibited higher decolorization efficiency than Lac4 for 11 out of 13 kinds of the tested dyes, which may attribute to the relatively higher catalytic efficiency of Lac3 than Lac4 (in terms of *k*_cat_/*K*_m_) towards syringaldazine and ABTS. It was reported that the crude laccases had more decolorization efficiency than purified laccase probably due to coexistence of different isoenzymes [43], [44]. However, there was no report to present synergistic interactions between purified laccase isoenzymes in decolorization previously. In this study, we observed the synergistic interactions between Lac3 and Lac4 in dye decolorization, but it is highly dyes dependent.

In conclusion, we successfully expressed two laccase isoenzymes from *C. comatu*s in *P. pastori*s by fusing with an additional ten amino acids tag at N-terminus. The Lac3 and Lac4 showed clearly different catalytic properties and dye decolorization abilities. So far, the biological functions of different isoenzymes in fungal species were still poorly understood. This study reinforced the laccase diversity in fungi and suggested that these laccase isoenzymes with a low sequence identity among individual members have diverse functions in *C. comatus*. The tolerance of Lac3 and Lac4 towards extreme conditions, including various metal ions and high concentration of SDS, as well as wide dye decolorization ability, demonstrated these laccase isoenzymes could be also potential candidates for biotechnological applications.

## Supporting Information

Figure S1 **Neighbor-joining tree of the deduced amino acid sequences of Lac3 and Lac4 and other laccases from GenBank.** The tree is calculated with p-distances using Mega ver. 5.2, based on a ClustalX alignment. Bootstrap values (1000 replications) higher than 50% are indicated at branchings.
(TIF)

Figure S2 **Detection of the recombinant Lac2 laccase activity on BMMY agar plates containing CuSO_4_ and ABTS (A) and the time course of extracellular Lac2 production in BMMY liquid medium (B).** A: transformants containing 1) pPICZαBB-Lac2, and 2) pPICZαB-10AALac2; B: transformants containing pPICZαB-10AALac2 (filled square), and pPICZαBB-Lac2 (filled circle).
(TIF)
