# Supplementary figures and images for: Engineering the Expression and Characterization of Two Novel Laccase Isoenzymes from Coprinus comatus in Pichia pastoris by Fusing an Additional Ten Amino Acids Tag at N-Terminus
Source: PLoS One. 2014 Apr 7;9(4):e93912. doi: 10.1371/journal.pone.0093912 (PMC3977997; doi:10.1371/journal.pone.0093912)

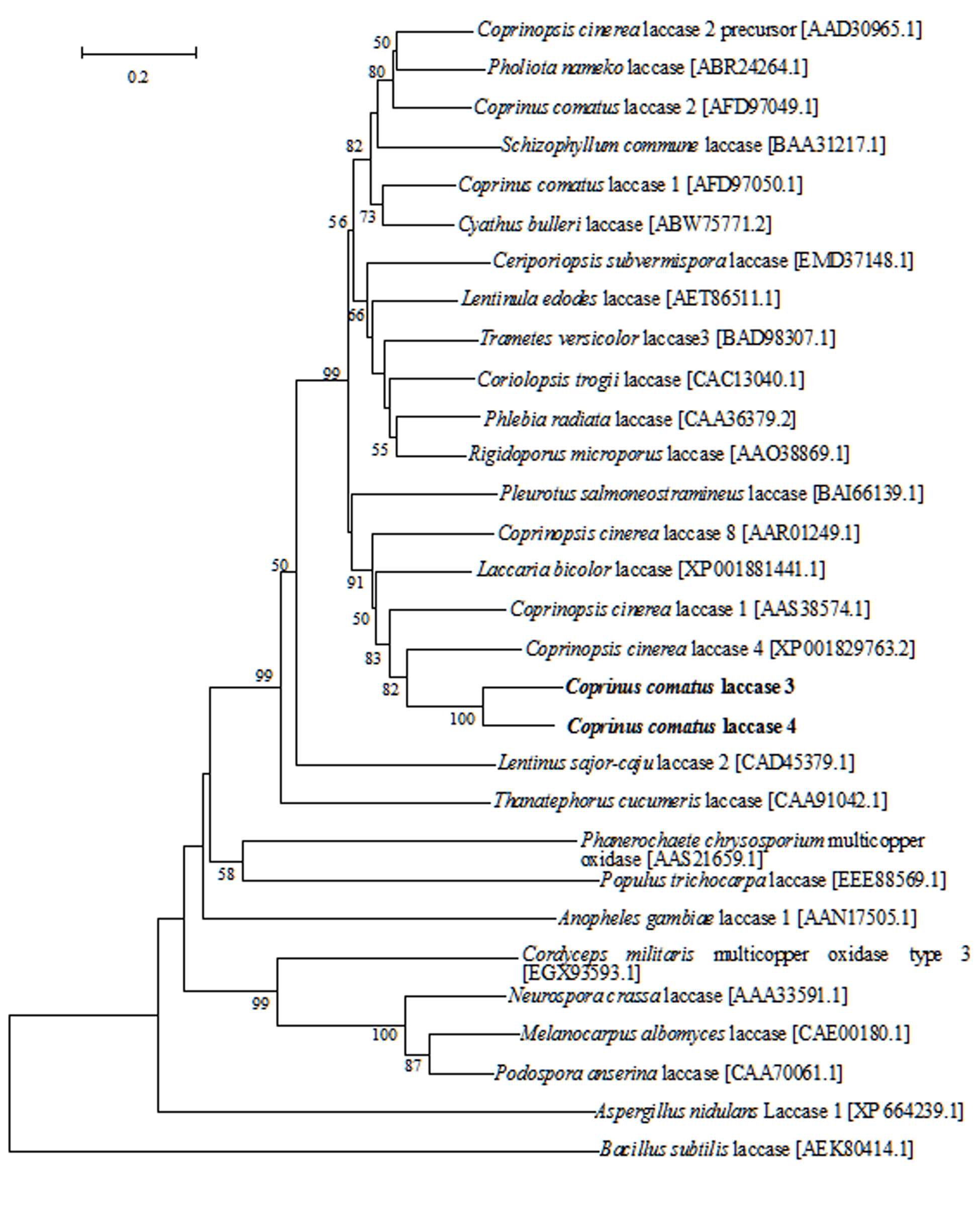

Supplement: Figure S1 — Neighbor-joining tree of the deduced amino acid sequences of Lac3 and Lac4 and other laccases from GenBank. The tree is calculated with p-distances using Mega ver. 5.2, based on a ClustalX alignment. Bootstrap values (1000 replications) higher than 50% are indicated at branchings. (TIF) [file pone.0093912.s001.tif]

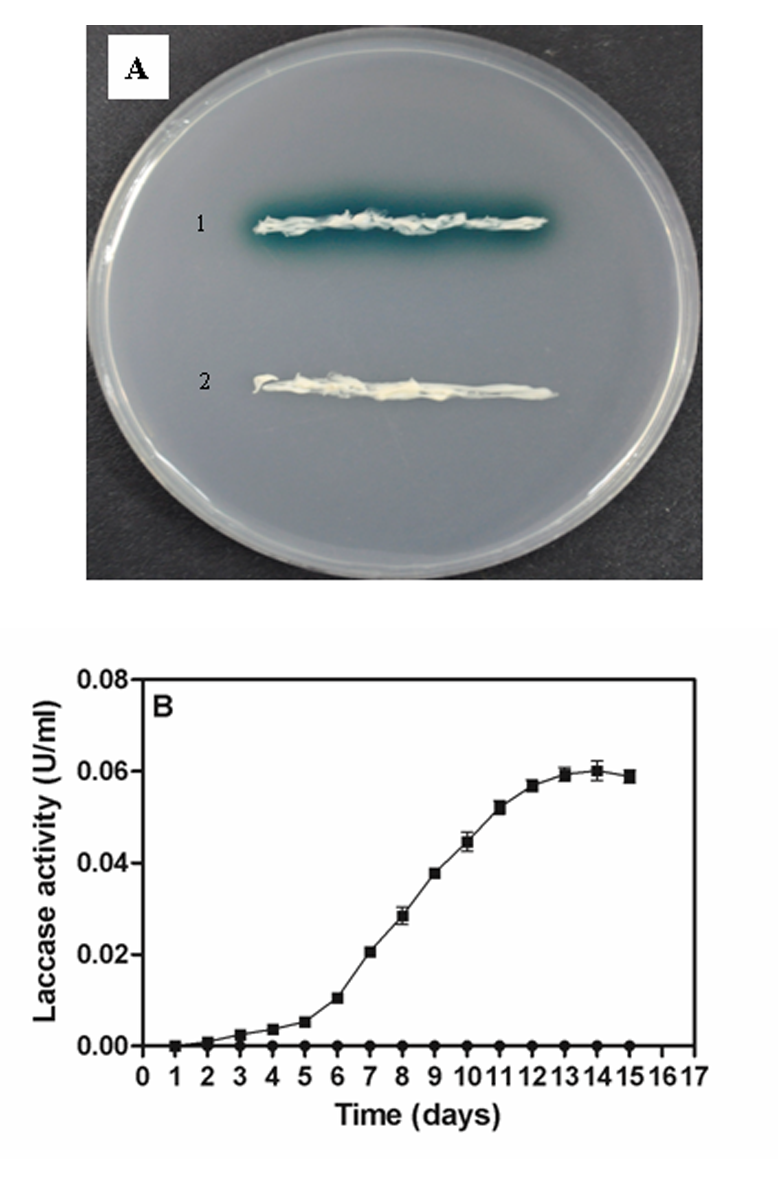

Supplement: Figure S2 — Detection of the recombinant Lac2 laccase activity on BMMY agar plates containing CuSO4 and ABTS (A) and the time course of extracellular Lac2 production in BMMY liquid medium (B). A: transformants containing 1) pPICZαBB-Lac2, and 2) pPICZαB-10AALac2; B: transformants containing pPICZαB-10AALac2 (filled square), and pPICZαBB-Lac2 (filled circle). (TIF) [file pone.0093912.s002.tif]
